# Successful intraoperative management of laparoscopic hysterectomy in a patient with Eisenmenger syndrome: a case report

**DOI:** 10.1186/s40981-024-00700-9

**Published:** 2024-03-04

**Authors:** Yuki Maeda, Nami Kakuta, Asuka Kasai, Hiroki Yonezawa, Ryosuke Kawanishi, Katsuya Tanaka

**Affiliations:** 1 Department of Anesthesiology, Tokushima University, 3-18-15, Kuramoto, Tokushima, 770-8503 Japan; 2 Division of Surgical Center, Tokushima University Hospital, Tokushima, Japan

**Keywords:** Eisenmenger syndrome, Laparoscopic surgery, General anesthesia

## Abstract

**Background:**

Patients with Eisenmenger syndrome (ES) requiring noncardiac surgery are at a significantly high risk of perioperative morbidity and mortality. However, perioperative management of patients with ES requiring laparoscopic surgery remains unclear.

**Case presentation:**

We describe the case of a patient with ES who underwent laparoscopic hysterectomy under general anesthesia with a peripheral nerve block. The objectives of the perioperative management included the following: (1) maintaining systemic vascular resistance and cardiac output through euvolemia, facilitated by the infusion of noradrenaline, and (2) preventing a reduction in oxygen-carrying capacity and factors that elevate pulmonary vascular resistance, such as pain, hypoxia, and decreased body temperature. Although laparoscopic procedures involved an increased risk in patients with ES, they are less invasive than open surgeries.

**Conclusion:**

This report describes the successful anesthetic management of a patient with ES, ensuring a balance between systemic and pulmonary vascular resistance.

## Background

Eisenmenger syndrome (ES) is a complex combination of cardiovascular abnormalities characterized by pulmonary hypertension at the systemic pressure level with a reversed or bidirectional shunt through intracardiac or aortopulmonary communication [1].

The mortality rate among patients with ES undergoing noncardiac surgery is as high as 3.8–30% [2, 3], and indications for surgery and anesthesia are limited [4]. During perioperative management, preventing an increase in pulmonary vascular resistance (PVR) and maintaining systemic vascular resistance (SVR) are essential [4]. This report describes our experience with anesthetic management of a patient with ES who underwent laparoscopic total hysterectomy under general anesthesia with a peripheral nerve block.

## Case presentation

A 34-year-old woman with ES was scheduled for laparoscopic total hysterectomy due to heavy bleeding during menstruation. Her weight, height, and body mass index were 73.3 kg, 1.62 m, and 28 kg/m^2^, respectively. She had a small ventricular septal defect at birth and was diagnosed with ES at the age of 7. She subsequently presented with severely limited exercise tolerance and began taking phosphodiesterase-5 inhibitors, prostacyclin receptor agonists, and endothelin receptor antagonists. In addition, she received home oxygen therapy. At 31 years of age, she was diagnosed with idiopathic thrombocytopenic purpura. Further, laboratory assessment revealed a mild prolongation of activated partial thromboplastin time. Since then, she had been hospitalized several times for hemoptysis and heavy menstrual bleeding.

Upon presentation to our hospital, the patient was in shock with a heart rate of 75–90 beats per minute (bpm) and arterial pressure of 86/54 mmHg due to severe bleeding. She developed progressive tachypnea; the oxygen saturation (SpO_2_) was around 80% during oxygen (O_2_) therapy using a simple face mask at a flow rate of 5 L/min. The laboratory findings were as follows: hematocrit level, 31.5%; platelet count, 10,000/µL; and activated partial thromboplastin time, 90.7 s. Arterial blood gas analysis yielded the following findings: pH, 7.445; PaO_2_, 47.8 mmHg; PaCO_2_, 29.3 mmHg; HCO_3_^−^, 19.7 mEq/L; and base excess, −3.5 mmol/L, with an O_2_ flow rate 5 L/min via a simple face mask. The patient also developed progressively worsening hypoxemia and cyanosis. After admission, she received a blood transfusion of red blood cells and fresh-frozen plasma, as well as intravenous immunoglobulin therapy. The perioperative team and her relatives discussed the risks of anesthesia and surgery and also considered nonsurgical management. Despite the related risks, a consensus was reached that hysterectomy was the best treatment alternative.

The patient underwent thorough preoperative assessment, including 12-lead electrocardiography (ECG), chest radiography, transthoracic echocardiography (TTE), full blood count, hepatic and renal function assessment, coagulation screening, and arterial blood gas analysis. Chest radiography revealed cardiomegaly, central pulmonary artery dilation, and disappearance of peripheral pulmonary vascular shadows. ECG revealed a sinus rhythm with right axis deviation and right ventricular hypertrophy. TTE demonstrated good left ventricular function; however, there was systolic displacement of the interventricular septum into the left ventricle, indicating right ventricular pressure overload. The peak gradient of tricuspid regurgitation increased markedly to 118 mmHg. The interventricular septum had a defect with a bidirectional shunt. Post-blood transfusion, blood values included the following: preoperative hemoglobin, 12.9 g/dl; hematocrit, 40.9%; platelet count, 101,000/µL; and activated partial thromboplastin time, 59.6 s.

A gynecologist recommended laparoscopy over open hysterectomy. Laparoscopic surgery involved a risk of exacerbation of the right heart failure due to changes in venous return and increased PVR due to pneumoperitoneum. Nevertheless, it was selected due to its benefits of minimal invasiveness, light pain, and faster recovery. We were prepared to switch to laparotomy if hemodynamics became unmanageable post-insufflation.

General anesthesia with peripheral nerve block was scheduled without any premedication. We used standard monitoring: ECG, pulse oximetry, end-tidal carbon dioxide partial pressure (P_ET_CO_2_), noninvasive blood pressure, and bispectral index monitor (BIS™, Medtronic, Minneapolis, MN, USA). A radial artery catheter was inserted before anesthesia, and the FloTrac sensor^®^ (ver.4.00, Edwards Lifesciences, LLC) were attached. Cardiac output (CO), cardiac index (CI), and stroke volume (SV) were evaluated using the HemoSphere monitor. Rapid anesthesia induction was achieved using 0.2 mg/kg of remimazolam, 100 µg of fentanyl, 50 mg of rocuronium, and continuous remifentanil infusion at 0.15 μg/kg/min. After tracheal intubation, transesophageal echocardiography (TEE) and central venous catheterization were performed. Additionally, rectus sheath and transversus abdominis plane blocks were administered for postoperative analgesia. Noradrenaline infusion at 0.02 μg/kg/min was started after the peripheral nerve block. Anesthesia was maintained using 1% sevoflurane and remifentanil up to 0.25 μg/kg/min, with bolus administration of rocuronium, as required.

The ventilation mode was pressure-controlled ventilation volume guaranteed (PCV-VG). Here, the target parameters were as follows: expired tidal volume, 6 mL/kg; inspiration-to-expiration ratio, 1:2; FiO_2_, 0.8 with air; positive end-expiratory pressure (PEEP), 5 cmH_2_O; and peak inspiratory pressure (PIP), ≤ 25 cmH_2_O. The P_ET_CO_2_ was maintained at 35–40 mmHg by adjusting the respiratory rate (RR). Intra-abdominal pressure during pneumoperitoneum was maintained at ≤ 8 mmHg, with a head down tilt of 15° being allowed.

**Fig. 1:**
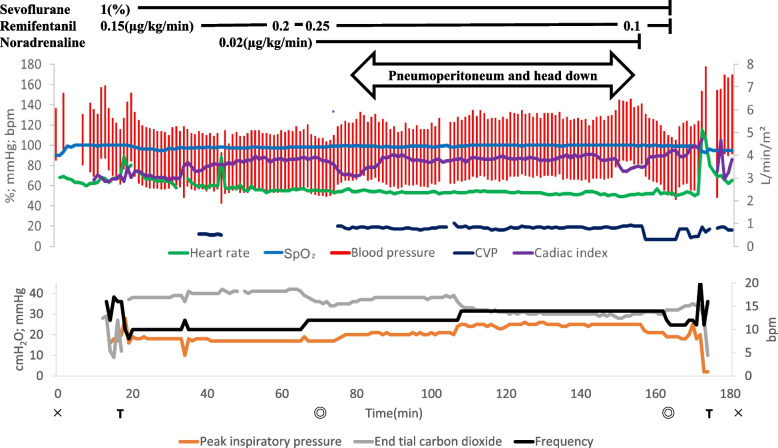
Anesthetic record. Parameters included are heart rate (bpm), saturation of percutaneous oxygen (SpO_2_; %), blood pressure (mmHg), central venous pressure (mmHg), cardiac index (L/min/m^2^), peak inspiratory pressure (cmH_2_O), end-tidal carbon dioxide (mmHg), and respiratory frequency (bpm). We thank you for your thoughtful suggestions and insights. The manuscript has benefited from these insightful suggestions

There were no acute episodes of hypotension, bradycardia, or arrhythmias during anesthesia induction or maintenance. Pneumoperitoneum or changing the patient’s position from supine to the head down position had no significant effect (Fig. 1). During pneumoperitoneum establishment, arterial blood gas analysis yielded normal findings (Table 1); blood pressure and heart rate remained unchanged, with the CI remaining at approximately 3.8 L/min/m^2^. Blood loss was 20 mL; accordingly, intraoperative blood transfusion was not required. The durations of surgery and anesthesia were 95 min and 183 min, respectively. The patient was subsequently extubated in the operating room.

**Table 1:** Results of sample analysis and results for FiO_2_, SpO_2_, and FECO_2_

Variable	Arterial (1)	Venous (2)	Arterial (3)	Venous (3)	Arterial (4)	Venous (4)
FIO_2_	1	0.8	0.8	0.8	1	1
SpO_2_ (%)	100	97	99	98	95	95
P_ET_CO_2_ (mmHg)		40	39	35		
Hemoglobin (g/dL)	13.2	12.4	12.3	12.3	12.4	12.4
pH	7.425	7.296	7.278	7.249	7.321	7.297
pCO_2_ (mmHg)	33.4	51.1	49.5	55.1	43.3	48.6
pO_2_ (mmHg)	118	50.7	117	51.2	80.2	42.3
HCO_3_^−^ (mmol/L)	22.9	22.3	21.2	21	21.3	21.4
BE (mmol/L)	−2.5	−1.6	−3.6	−3.1	−3.7	−2.7
SO_2_/ScvO_2_ (%)	99.7	80.6	98.9	79.5	95.8	72.4

Arterial samples were obtained from the radial artery, while venous samples were drawn from the central vein. 1, before induction of anesthesia; 2, after tracheal intubation; 3, after insufflation; 4, after tracheal extubation

*FiO*_*2*_ Fraction of inspired oxygen, *PCO*_*2*_ Partial pressure of carbon dioxide, *PO*_*2*_ Partial pressure of oxygen, *BE* Base excess, *SO*_*2*_*/ScvO*_*2*_ Blood gas oxygen saturation

The patient was transferred to the intensive care unit and could breathe spontaneously through a face mask with an O_2_ flow rate of up to 6 L/min; SpO_2_ was generally maintained at ≥ 90%. No episodes of hypotension occurred during the postoperative period. The patient received fentanyl infusion for 12 h at 25–50 µg/h. Analgesia was supplemented with intravenous acetaminophen. Oral administration of pulmonary vasodilators was resumed immediately on the day of surgery. The patient recovered uneventfully and was discharged 6 days postoperatively.

## Discussion

The primary goal of anesthetic management in patients with ES is to maintain SVR, minimize increases in PVR, and provide adequate analgesia [4]. Laparoscopic surgery causes unique hemodynamic and ventilatory effects [5], but it is less invasive and has a smaller effect on postoperative hemodynamics. Thus, it may be beneficial for patients with ES if appropriate anesthesia management is performed.

Changes in hemodynamics caused by abdominal insufflation include increase in MAP, SVR, and CVP and decrease in CO [5]. Among these, increased MAP and SVR may be advantageous in managing anesthesia for patients with ES. On the other hand, excessive preload on the right heart system and decreased CO can lead to a shunt crisis. However, regarding CO, the Trendelenburg position and intra-abdominal pressure below 15 mmHg can prevent a decrease in CO after abdominal insufflation [5]. The desufflation phase of laparoscopic surgery has the opposite effect to the insufflation phase on hemodynamics. To visualize these hemodynamic changes, we used the FloTrac sensor^®^, TEE, and central venous catheters. By monitoring, we confirmed stable hemodynamics throughout the entire surgery that progressed as expected from preoperative estimates. However, a decrease in ScvO_2_ was observed after extubation, which was thought to be caused by a decrease in SaO_2_ and an increase in oxygen consumption. Fortunately, hemodynamic failure did not occur; however, it is necessary to extubate patients with ES more carefully. Continuous ScvO_2_ measurement may also be desirable for longer or more invasive surgeries in patients with ES.

There are several reports on the continuous administration of noradrenaline to maintain systemic blood pressure in anesthesia management of patients with ES [6]. Noradrenaline, with α- and mild β-adrenergic activity, minimally affects heart rate and boosts CO [7]. It was also useful in maintaining CO and systolic blood pressure during anesthesia in this patient. Because excessive afterload leads to a decrease in CO, continuous administration of noradrenaline was stopped immediately before the end of surgery, but no significant decrease in systolic blood pressure was observed. Sammut et al. reported continuous administration of noradrenaline at 0.03–0.1 µg/kg/min during surgery and 0.03 µg/kg/min for 20 h postoperatively [6]. This patient received a continuous administration of 0.02 µg/kg/min during surgery, and we judged it unnecessary after surgery, although careful observation was still necessary.

Increased PVR can precipitate a shunt crisis. In particular, laparoscopic surgery can lead to hypoxemia and hypercarbia in patients with ES, requiring careful respiratory management. Therefore, ventilator management of patients with ES should emphasize higher FiO2, hyperventilation, *PEEP* < 15 mmHg (preferably between 5 and 10 cmH_2_O), and maintenance of the lung volume at a normal functional residual capacity [8]. To achieve this, our patient was intubated and received positive-pressure ventilation. The PCV-VG ventilation mode resulted in a decrease PIP and increased dynamic compliance compared to the volume-controlled ventilation (VCV) mode [9]. However, positive-pressure ventilation can suppress venous return, so early tracheal extubation was performed in the operating room.

Excessive tone of the sympathetic nervous system due to pain can cause an increase in SVR and PVR, followed by destabilization hemodynamics. Therefore, appropriate analgesia in the perioperative period is important. We administered multimodal analgesia including peripheral nerve block and were able to obtain sufficient postoperative analgesia. Peripheral nerve blocks can diminish the need for high-dose postoperative opioids, which are useful in avoiding postoperative hypoxemia and hypercapnia.

We believe that with proper anesthesia management, laparoscopic surgery can be a viable option for noncardiac surgery in patients with ES.

## Data Availability

Not applicable due to patient privacy concerns.

## Abbreviations

CI: Cardiac index
CO: Cardiac output
ECG: Electrocardiography
ES: Eisenmenger syndrome
ICU: Intensive care unit
PCV-VG: Pressure-controlled ventilation volume guaranteed
PEEP: Positive end-expiratory pressure
P_ET_CO_2_: End-tidal carbon dioxide partial pressure
PIP: Peak inspiratory pressure
PVR: Pulmonary vascular resistance
RR: Respiratory rate
SV: Stroke volume
SVR: Systemic vascular resistance
TEE: Transesophageal echocardiography
TTE: Transthoracic echocardiography
VCV: Volume-controlled ventilation
